# Growth and Maintenance of *Wolbachia* in Insect Cell Lines

**DOI:** 10.3390/insects12080706

**Published:** 2021-08-06

**Authors:** Ann M. Fallon

**Affiliations:** Department of Entomology, University of Minnesota, 1980 Folwell Ave., St. Paul, MN 55108, USA; fallo002@umn.edu

**Keywords:** *Wolbachia*, alpha-proteobacteria, reproductive parasite, symbiont, mosquito, insect cell lines, genetic manipulation, cell culture

## Abstract

**Simple Summary:**

*Wolbachia* is an intracellular bacterium that occurs in arthropods and in filarial worms. First described nearly a century ago in the reproductive tissues of *Culex pipiens* mosquitoes, *Wolbachia* is now known to occur in roughly 50% of insect species, and has been considered the most abundant intracellular bacterium on earth. In insect hosts, *Wolbachia* modifies reproduction in ways that facilitate spread of the microbe within the host population, but otherwise is relatively benign. In this “gene drive” capacity, *Wolbachia* provides a tool for manipulating mosquito populations. In mosquitoes, *Wolbachia* causes cytoplasmic incompatibility, in which the fusion of egg and sperm nuclei is disrupted, and eggs fail to hatch, depending on the presence/absence of *Wolbachia* in the parent insects. Recent findings demonstrate that *Wolbachia* from infected insects can be transferred into mosquito species that do not host a natural infection. When transinfected into *Aedes aegypti*, an important vector of dengue and Zika viruses, *Wolbachia* causes cytoplasmic incompatibility and, in addition, decreases the mosquito’s ability to transmit viruses to humans. This review addresses the maintenance of *Wolbachia* in insect cell lines, which provide a tool for high-level production of infectious bacteria. In vitro technologies will improve use of *Wolbachia* for pest control, and provide the microbiological framework for genetic engineering of this promising biocontrol agent.

**Abstract:**

The obligate intracellular microbe, *Wolbachia pipientis* (Rickettsiales; Anaplasmataceae), is a Gram-negative member of the alpha proteobacteria that infects arthropods and filarial worms. Although closely related to the genera *Anaplasma* and *Ehrlichia*, which include pathogens of humans, *Wolbachia* is uniquely associated with invertebrate hosts in the clade Ecdysozoa. Originally described in *Culex pipiens* mosquitoes, *Wolbachia* is currently represented by 17 supergroups and is believed to occur in half of all insect species. In mosquitoes, *Wolbachia* acts as a gene drive agent, with the potential to modify vector populations; in filarial worms, *Wolbachia* functions as a symbiont, and is a target for drug therapy. A small number of *Wolbachia* strains from supergroups A, B, and F have been maintained in insect cell lines, which are thought to provide a more permissive environment than the natural host. When transferred back to an insect host, *Wolbachia* produced in cultured cells are infectious and retain reproductive phenotypes. Here, I review applications of insect cell lines in *Wolbachia* research and describe conditions that facilitate *Wolbachia* infection and replication in naive host cells. Progress in manipulation of *Wolbachia* in vitro will enable genetic and biochemical advances that will facilitate eventual genetic engineering of this important biological control agent.

## 1. Introduction

*Wolbachia* is an obligate intracellular microbe first described in reproductive tissues of *Culex pipiens* mosquitoes nearly a century ago [1,2]. Like *Escherichia coli*, *Wolbachia* is a Gram-negative bacterium in the phylum Proteobacteria: the purple bacteria and their relatives. Proteobacteria include nine monophyletic classes representing tremendous biodiversity. Among these, the genera *Ehrlichia* and *Anaplasma*, which can cause disease in humans, are classified with *Wolbachia* as members of the alpha-proteobacteria, in the order Rickettsiales, family Anaplasmataceae. *Wolbachia* is uniquely associated with invertebrates, does not infect vertebrate hosts, and replicates only within a eukaryotic host cell. In contrast, *E. coli* and many familiar Gram-negative pathogens of humans classified as gamma-proteobacteria can be cultured in liquid medium and plated on solid media as free-living microbes.

Knowledge of well-studied free-living bacteria provides an important framework for investigating the genetics and physiology of *Wolbachia*, now known to infect a high proportion of insect species, in addition to other arthropods and filarial worms, all members of the Ecdysozoa. Because of its widespread distribution among insects [3,4], *Wolbachia* provides a model system for exploring biological interactions between an intracellular microbe, the invertebrate host cells in which it resides, and the diversity of reproductive phenotypes with which it is associated [5,6]. In species that harbor *Wolbachia,* the bacterium is transmitted vertically, from mother to offspring, which retain the infection. In most arthropods, *Wolbachia* alters reproduction in diverse ways that favor its invasion of naive populations, and is sometimes considered a reproductive parasite. In contrast, *Wolbachia* is an essential symbiont in filarial worms [7,8,9]. In mosquitoes, *Wolbachia* causes a reproductive distortion called cytoplasmic incompatibility (CI), which has important applications in vector control [10].

## 2. A Brief History of *Wolbachia* and Cytoplasmic Incompatibility in Mosquitoes

The species name for *Wolbachia, Wolbachia pipientis*, reflects its discovery in reproductive tissues of the mosquito *Cx. pipiens* [1,2]. *Wolbachia* was described during the historical period when arthropod-borne intracellular bacteria were first appreciated as pathogens that cause disease in humans. Notable discoveries during that time included those of Howard Ricketts, who observed that a tick-borne bacterium, now known as *Rickettsia rickettsii*, was the causative agent of Rocky Mountain Spotted Fever [11], and Henrique da Rocha Lima, who described *Rickettsia prowazekii* as the cause of epidemic typhus [12]. Simeon Burt Wolbach was involved in identification of the louse as the vector of typhus [13], and it is not surprising that he noticed similarities between arthropod-borne pathogens and the intracellular bacteria now known as *Wolbachia*. Within the Rickettsiales, members of the genus *Rickettsia* are now assigned to the family Rickettsiaceae (short rods or coccobacilli), and *Wolbachia*, to the family Anaplasmataceae (small pleomorphic cocci); these families have been distinguished based on genetic analyses [14]. For many years, *Wolbachia* was thought to be unique to *Cx. pipiens* mosquitoes because it appeared to be restricted to reproductive tissues, whereas other microbial symbionts in arthropods were more widely distributed among host tissues [1,2]. In retrospect, however, it should be noted that methods for distinguishing species of intracellular microbes in insects were poorly developed at that time.

The best-studied effect of *Wolbachia* is the reproductive distortion known as cytoplasmic incompatibility (CI). Discovery of CI was a fortuitous result of the fact that *Cx. pipiens* mosquitoes mate in small cages and establish breeding populations under laboratory conditions. In studies unrelated to the microbiology of *Wolbachia*, crosses between *Cx. pipiens* from independent laboratory colonies representing diverse geographic regions sometimes exhibited the peculiar, maternally inherited mating distortion called CI. CI was manifested when eggs from crosses between male and female mosquitoes originating from different regions failed to hatch, in a pattern eventually defined by 17 distinct cytotypes [15,16,17,18]. Even before the connection between CI and *Wolbachia* was established, maternal inheritance of CI was recognized as a gene drive mechanism potentially useful for population replacement of vector mosquitoes [19,20]. The association between *Wolbachia* and CI was established when Yen and Barr demonstrated reversal of the CI phenotype in *Cx. pipiens* after treatment with antibiotics, which eliminated the bacterium [21,22]. The molecular biology and biochemistry of CI and the genes associated with this phenotype in *Drosophila* and mosquitoes have been reviewed, and will not be discussed in detail here [17,18].

## 3. Contemporary Wolbachia Research

Development of genomic sequencing and related molecular technologies, and the discovery of *Wolbachia* sequences in DNA samples from *Drosophila melanogaster* and other insects, contributed substantially to our understanding of the biology, distribution, and diversity of *Wolbachia*. Advancement of *Wolbachia* research through the use of *D. melanogaster* mutants is only beginning and, among mosquitoes, the presence of *Wolbachia* in *Aedes albopictus*, and its absence in important vectors such as *Aedes aegypti* and *Anopheles gambiae*, has provided an important incentive for *Wolbachia* research. *Wolbachia* also occurs in agricultural pests, including planthoppers that feed on rice, and lepidopteran pests of food crops.

Publication of the first *Wolbachia* genome from strain *w*Mel infecting *D. melanogaster* revealed a streamlined, AT-rich genome of 1.27 Mbp encoding 1144 proteins (NC_002978.6), compared to the *E. coli* genome of 4.64 Mbp, 4242 proteins for K-12 sub-strain MG1655 (NC_000913.3). The *w*Mel genome contains a putative prophage/s and an unusual abundance of mobile elements, relative to genomes from other intracellular bacteria [23]. Comparative analysis of *Wolbachia* genomes has provided important insights into the correlations between gene loss, symbiosis, *Wolbachia*’s dependence on its host for essential nutrients, and its genetic capabilities; complete or nearly-complete sequence annotation for several representative genomes is available on the NCBI website (ncbi.nih.nlm.gov).

As noted above, a *Wolbachia* isolate is typically called a strain, and is usually named for its host species; for example, *Wolbachia* from *D. melanogaster* is called *w*Mel; that from *Cx. pipiens* is known as *w*Pip. Individual strains are further subdivided into 17 monophyletic clusters of diversity, or supergroups, designated by letters [24,25]. As more strains are described, nomenclature based on species names of hosts is becoming cumbersome. For example, *w*Ctub could refer to a supergroup J *Wolbachia* from a filarial worm [26] or a supergroup F *Wolbachia* from a termite [27].

Consistent with the wide diversity of its hosts, *Wolbachia* infections are associated with phenotypes other than CI, including parthenogenesis, male killing, and feminization of genetic males, which are not necessarily uniform among members of a supergroup. Like CI, these phenotypes have a net effect of increasing the abundance of *Wolbachia* in host populations [5,6,28] and, in many cases, the arthropod host can be cured of the *Wolbachia* infection, with loss of associated phenotypes, by treatment with rifampicin and/or tetracycline [29]. Filarial worms, and a few arthropods including the bedbug *Cimex lectularius* (Hemiptera), the wasp *Asobara tabida* (Hymenoptera), and the springtail *Folsomia candida* (Collembola), in which *Wolbachia* has become a symbiont, are refractory to antibiotic cure. In humans, antibiotics have therapeutic value for reducing filarial infection [9].

Although reproductive phenotypes can be validated by comparing infected relative to antibiotic-cured populations, it has been difficult to document *Wolbachia*’s more general effects on the biology and ecology of its hosts [30]. Evaluation of host fitness under laboratory conditions is subject to complex variables [31], and although both positive and negative effects have been described, the narrative has been confounded by undefined contributions of the host’s genetic background [31,32,33]. Similarly, unpublished comparisons between infected and tetracycline-cured *Cx. pipiens* adults in the author’s laboratory have thus far yielded inconsistent results that may reflect, at least in part, variability in larval rearing conditions. *Wolbachia*’s individual associations with diverse hosts may preclude broad generalizations, and the complexity of *Wolbachia*’s interactions with host tissues provides a rich field for further investigation. As described below, fitness issues have become particularly important in efforts to establish novel *Wolbachia* associations for control of mosquito vectors.

Present day interest in *Wolbachia* as an environmentally-friendly approach to vector control builds upon early successful demonstrations that its associated gene drive can be harnessed for vector replacement [19,20,34], coupled with more recent methods for rapid detection of *Wolbachia* infections; successful introduction (transinfection) into non-host species such as the dengue vector, *Ae aegypti*, and the malaria vector, *Anopheles gambiae* [10]; retention of the CI phenotype and maternal transmission after transinfection; and, in mosquitoes, the unanticipated suppression of pathogen transmission [10,35,36,37]. Additional biological factors, including fecundity, hatch rate, diapause survival, longevity, *Wolbachia* density, and tissue distribution, and virus-blocking capacity, are similarly important for successful *Wolbachia*-based control applications. Several ongoing studies address optimization of *Wolbachia* to control disease transmission, including combination with sterile insect techniques [38]. Although such applications are underway, much remains to be learned about the basic biology of *Wolbachia*, and the potential for its genetic modification using technologies that have become routine for *E. coli*.

## 4. *Wolbachia* in Insect Cell Lines

*Wolbachia*’s obligate intracellular lifestyle complicates the biochemical and genetic analyses that could advance pest control and anti-filarial applications. Even with hosts amenable to laboratory rearing, maintenance of colonies, dissection of infected tissues, and embryonic microinjection are labor-intensive and time-consuming. Moreover, many existing laboratory colonies are highly inbred, complicating cage studies that address fitness. The utility of *Wolbachia* in control applications would be enhanced if the microbe could be experimentally manipulated by genetic engineering to express selectable markers, which in turn will be advanced by improving manipulation of *Wolbachia* in cell lines and expanding the diversity of *Wolbachia* strains that can be investigated in culture. A modest advance would be adaptation of a filarial strain of *Wolbachia* to a cell line; at present, *Wolbachia*-infected insect cell lines are used as a surrogate to identify new drugs that target *Wolbachia* for treatment of filarial diseases [39,40,41].

The author’s research focuses on systematic exploration of *Wolbachia* propagation in cultured cells as a substitute for the differentiated host tissues, such as ovaries and testes, in which *Wolbachia* is most abundant. Cell lines used to propagate *Wolbachia* are listed in Table 1, wherein supergroup designations are noted after the strain name; for example, *w*Pip_B indicates that *w*Pip is classified in supergroup B. With the exception of a single member of supergroup F from the cat flea [42], only members of supergroups A and B, sometimes called the “pandemic” supergroups, have been maintained in insect cell lines. The reader should note that, in some cases, an infected cell line may have been sub-cultured only a limited number of times and/or has a very long doubling time, and that the same cell line may have been infected with the same strain of *Wolbachia* by different investigators, and given a different name. An important incentive for employing cell lines was the possibility that preadaption to cultured cells might improve the likelihood that *Wolbachia* would establish in novel hosts infected by embryonic microinjection, and towards this end, a few lines have been maintained for several years [43]. In other cases, which are not reviewed in detail here, infected cell lines have been used to test effects of *Wolbachia* on viral replication in efforts that generally validate the anti-pathogen responses seen in transinfected mosquitoes. Finally, as with *Wolbachia* itself, a uniform descriptive label for infected cell lines remains to be developed.

## 5. Cell Lines Derived from Infected Insects

In Table 1, two types of *Wolbachia*-infected cell line are noted: those established directly from infected tissues, which retain *Wolbachia* from the host, and lines established from uninfected cells into which *Wolbachia* has been artificially introduced, either from infected tissues or from a previously infected cell line. O’Neill and coworkers were the first to describe a line with a host-derived *Wolbachia* infection, which they named Aa23 [44]. Aa23 cells are persistently infected with *w*AlbB, one of two strains that co-infected the host *Ae. albopictus* mosquitoes. Curiously, at least in some geographic areas, when a single *Wolbachia* strain is found in *Ae. albopictus*, it types to strain *w*AlbA [70]. Given that *Aedes albopictus* hosts a natural infection, one might wonder why *Wolbachia* is absent from older *Ae. albopictus* lines dating back to the late 1960s, before *Wolbachia* was known to be widespread in insects [63]. The possibility exists that early in their history, such lines may have been infected, followed by loss of *Wolbachia* over decades of use in multiple laboratories.

Other cell lines developed directly from infected hosts include JW-18, which carries *w*MelPop from *D. melanogaster* [41]. In addition, a *Wolbachia* strain in a primary culture called Dm2008Wb1, from which the *Wolbachia* was subsequently transferred to *Drosophila* S2 cells, has been developed in Russia [65]. Although it can take considerable time to establish primary cultures, which require additional time to become established, it will be of interest to learn whether primary cultures established directly from carefully chosen *Drosophila* mutants will provide insights into *Wolbachia*’s interactions with host cells.

Extrapolation of *Wolbachia*’s behavior in host tissues to cultured cells is not straightforward; similarly, it is not known how cultured cells should be manipulated to favor successful establishment of a persistent infection. For example, studies with *Ae. albopictus* eggs suggest that the highest *Wolbachia* densities occur during embryogenesis, and that replication is dependent on host cell division [71], whereas *w*MelPop replicates best in postmitotic cells of adult flies [72]. How established infections coevolve with host cells and potentially modify the host cell cycle are yet to be explored. Methods for distinguishing live *Wolbachia* from metabolically active and/or infectious *Wolbachia* are not yet available, and will be needed to determine whether maintenance of *Wolbachia* in one cell line facilitates its establishment in a second cell line, or whether the initial cell line simply provides a higher multiplicity of infectious particles. When examined by fluorescence microscopy, a single *Cx. pipiens* ovary appears to contain innumerable *Wolbachia*, but it is not known whether all are equally infectious, whether the proportion of microbes capable of initiating infection changes during the mosquito reproductive cycle, and what factors define the small proportion of *Wolbachia* that establish in the embryonic germline. Finally, it will be of interest to learn whether *Wolbachia* (or other endogenous infectious agents such as insect-specific flaviviruses) influence how easily a permanent cell line can be established from a host species. Compared to those from *Ae. albopictus* and *Ae. aegypti* mosquitoes, relatively few established cell lines are available from *Cx pipiens* [63,73].

## 6. Transfers between Cell Lines

As with infected insects, *w*AlbB can be removed from Aa23 cells by treatment with tetracycline, and these cured Aa23(T) cells have been a popular host for establishment of new infections [43,45,46]. Indeed, the majority of cell lines into which *Wolbachia* has been introduced derive from *Ae. albopictus*, and the possibility that these successes are influenced by host factors that coevolved with the natural *Wolbachia* infection in ancestral mosquitoes has not been addressed. Note, for example, that the RML-12 cell line, originally used to “preadapt” *Wolbachia* for transfer to *Ae. aegypti*, is now known to have originated from *Ae. albopictus* [43,45]. Aa23(T) cells host the single example of *w*Pip in culture [46]. Despite the importance of *w*Pip as one of the best-understood models for CI [74,75,76], this author has been unable to establish *w*Pip in culture, and is not aware of a cell line that maintains a long-term persistent infection with *w*Pip.

One of the most interesting *Wolbachia* strains introduced into Aa23(T) cells is *w*MelPop, an unusually virulent strain isolated from *D. melanogaster*. Briefly, to investigate brain degeneration in *Drosophila*, Min and Benzer crossed an X-chromosome deficiency strain with reduced life span into the *white* mutant *w^1118^,* which had a normal life span [72]. Resulting progeny expressed the short life span, which was associated with a maternally-transmitted bacterium identified as the “*popcorn*” strain of *Wolbachia. Popcorn* proliferates primarily in postmitotic tissues of the adult, causing death of the host fly. Despite its ovarian transmission, *popcorn* does not cause CI in the original *Drosophila* strain described by Min and Benzer [72], but the cell-line adapted variant does cause CI in *Drosophila* [43] and in transinfected mosquitoes [10]. Genome comparisons suggest that virulence of *w*MelPop is caused by copy number variation of a 21 kb “octomom” region encoding eight genes of unknown function [77,78].

The strain called *w*MelPop-CLA designates a “cell-line adapted” version of *w*MelPop, created by infecting Aa23(T) cells with *w*MelPop from *w^1118^* eggs [43]. Although the rationale for adapting *w*MelPop to a cell line related to difficulties in using *Wolbachia* from infected tissues directly to establish a stable transovarial infection in a novel host, transfer of *Wolbachia* into an insect cell line can also be problematic. For example, establishment of *w*MelPop in Aa23(T) cells was successful in only two of 68 attempts [43]. After adaptation to mosquito cells, *w*MelPop-CLA was reintroduced into *Drosophila* embryos, in which it exhibited a diminution, but not complete loss, of properties related to life shortening, bacterial density, and CI, relative to the parental strain [43]. The life-shortening effect of *w*MelPop was of interest because reduced longevity of vector species would be expected to reduce pathogen transmission [79], but *w*MelPop-CLA, and to a lesser extent *w*Mel, have deleterious effects on *Ae. aegypti* eggs and larval development [35,80], which have remained stable, at least over 10 years [81]. Other mosquito cell lines that maintain *w*MelPop-CLA include RML-12 (*Ae. albopictus*) and Mos-55 (*An. gambiae*).

A third variant from *D. melanogaster*, *w*MelCS, was isolated from Canton S flies [78]. Genomic studies indicate that *w*MelCS arose prior to *w*Mel, and is ancestral to *w*MelPop. In nature, *w*Mel appears to be replacing *w*MelCS [82]. The extent to which the *Wolbachia* genome undergoes changes during growth in cultured cells has been evaluated in a genetic comparison of *w*Mel strains, in which five genetic differences were detected after introduction into cell lines: an IS5 insertion, a multi-gene deletion, two point mutations, and a 10 bp deletion [78]. Because comparable changes did not occur with cell line-derived *Wolbachia* transinfected into mosquitoes, it is possible that these genetic changes were triggered by cross-species transfer. Nevertheless, the changes are not known to affect essential genes or cause major genomic rearrangements, suggesting to a first approximation that use of genetically manipulated *Wolbachia* produced in cell lines will remain suitable for control applications. Although genome evolution of tissue-derived *Wolbachia* in cross-species transfers has not yet been investigated, recent in-depth comparisons suggest that relative to *w*Ri (from *Drosophila simulans*) and *w*Pip (from *Cx. pipiens* mosquitoes), *w*MelCS appears to be the best candidate for transinfection into *Ae. aegypti* for field release [35].

The *Ae. albopictus* cell line NIAS-AeAl-2 supports the Hemipteran *Wolbachia* strain, *w*Stri, which was transferred from ovaries of the planthopper *Laodelphax striatellus*; this line also supports lepidopteran strains *w*Kue, *w*CauA, and *w*CauB [47,48]. More recently, we found that *w*Stri establishes a particularly robust infection in clonal C7-10 *Aedes albopictus* cells [53], and used the resulting C/*w*Stri1 line to develop flow cytometric quantitation of *Wolbachia* infections [83]. The *w*Stri strain also replicates in tick cell lines [42]. Strain *w*CauA provides an interesting model for investigating a single *Wolbachia* strain that causes two distinct phenotypes, CI and male killing, in different hosts [84,85], a phenomenon also described in *Drosophila subquinaria* [86]

## 7. *Aedes aegypti* Cells and New Viruses

Aside from a single transfer into *Ae. aegypti* Aa20 (Mos-20) cells [62,63], the Aag2 line is most commonly used in *Wolbachia* studies, including many that focus on *Wolbachia*’s antiviral effects. Aag2 cells are a subpopulation of cells isolated in the Peleg laboratory (Israel Institute for Biological Research, Ness-Ziona, Israel) that we adapted to Eagle’s medium [87] and designated Aag2 because the precise identity of Peleg’s culture (line 59 vs. line 364) was uncertain. Metaphase spreads from Aag2 cells contain a characteristic chromosome fragment, and the cells can be distinguished from *Ae. albopictus* cells by electrophoretic mobility of small heat shock proteins. Aag2 cells host an inapparent infection with the insect-specific flavivirus called Cell Fusing Agent (CFA), which was discovered when *Ae. aegypti* cells were co-cultured with *Ae. albopictus* cells [88]. For virological studies, this endogenous virus made *Ae. aegypti* cells less desirable, relative to *Ae. albopictus* cells, for classical studies with mosquito-borne viruses [89,90]. Although the generation of the clonal C7-10 and C6/36 lines included steps to exclude viruses [89], more recent proteomic studies do not exclude the presence of endogenous viruses in C7-10 cells [91].

In Aag2 cells, *Wolbachia* inhibits CFA replication [92], in addition to the replication of a newly described positive-sense RNA negev-like virus [57], but the antiviral effect may differ in field populations of mosquitoes [93]. More recently, a bunyavirus called Phasi Charoen-like virus was also found persistently to infect Aag2 cells, but is not inhibited by *Wolbachia*, possibly reflecting its negative-sense RNA genome [61,94]. Similarly, a negative-sense RNA anphevirus found in *Aedes* cell lines and mosquitoes is not inhibited by *Wolbachia* [95].

## 8. Other Dipteran, Lepidopteran and Tick Cell Lines

Newly established dipteran cell lines that support *Wolbachia* include those derived from the sandfly, *Lutzomyia longipalpis*, the biting midge, *Culicoides sonorensis*, and the horn fly, *Hematobia irritans*. Two lepidopteran lines, from the moths *Heliothis zea* and *Spodoptera frugiperda*, support *w*Stri, *w*Ri, and *w*CauB, whereas cell lines from the ticks *Ixodes scapularis*, *Ixodes ricinus*, and *Riphicephalus microplus* support *w*AlbB and *w*Stri. Uniquely, establishment of *w*Cfe_F in *Ixodes scapularis* lines raises the expectation that members of supergroups other than A and B will eventually be cultured in vitro [42]. Filarial strains of *Wolbachia* have not yet been cultured; by default, infected mosquito cell lines have been used as surrogates to screen for potential anti-filarial drugs [39,40,41].

## 9. Studies with *w*AlbB and *w*Stri in C7-10 Cell Derivatives

My own lab has worked extensively with the *Aedes albopictus* C7-10 cell line [89,96,97] and *Wolbachia* strains *w*AlbB [44,55] and *w*Stri [47,53,56]. C7-10 is a clonal population developed as a standard “wild-type” cell line adapted to a modified Eagle’s medium that lacks undefined components, such as yeastolate or lactalbumin hydrolysate, that are included in many insect-specific media formulations. Because Eagle’s medium has a defined chemical composition, apart from the supplemental serum, it facilitates use of the techniques and approaches of somatic cell genetics to isolate viral and host cell mutants [90,96,97]. This medium is bicarbonate-buffered, and cells are maintained at 28 to 30 °C in a 5% CO_2_ atmosphere. The persistently-infected line called C/*w*Stri1 was produced by inoculating *w*Stri from NIAS-AeAl-2 cells [47] into a growing population of C7-10 cells [53].

Although C/*w*Stri1 cells are primarily used in current studies, Aa23 cells infected with *w*AlbB provided the starting point for our investigations [98]. Aa23 cells represent a mixed cell population from embryonic tissue that maintains a somewhat variable level of *w*AlbB in individual cells and between populations of cells [44]. In our hands, Aa23 cells readily adapted to Eagle’s medium containing 20% heat-inactivated fetal bovine serum. In contrast to clonal C7-10 cells, Aa23 cells grew as patchy monolayers that tended to form solid aggregates as cultures aged; these cytological characteristics persisted after elimination of *Wolbachia* with tetracycline [98]. Because the solid clusters reach sizes visible to the naked eye, it seemed likely that cells within a cluster vary in metabolic activity as direct contact with the culture medium decreases. In addition, it seemed possible that internal cells may die and/or be cannibalized by cells growing closer to the surface of the cluster. To minimize cell clusters, we often used trypsin and/or passed resuspended cells through a 40 μm nylon mesh before plating. Although additional isolates of Aa23 lines were available in liquid nitrogen storage in the lab of Dr. Robert Tesh (University of Texas Medical Branch, Galveston, TX, USA), inspection of these lines (Fallon, 2005, unpublished) suggested that none was more tractable than the Aa23 line described by O’Neill and coworkers [44]. It remains to be learned whether the tendency of Aa23 cells to aggregate is relevant to recovery of a *Wolbachia*-infected line, and whether the desirability of cell lines that grow as attached, relatively uniform monolayers may have contributed to inadvertent loss of infection with continued passage of long-established *Ae. albopictus* cell lines.

Aa23 cells grow relatively slowly with a doubling time of 4–5 days, compared to 20 h with C7-10 cells. Counting Aa23 cells using a Coulter electronic cell counter can be facilitated by adding NP-40 to the culture medium to release cell nuclei, rather than attempting to disrupt clusters of tightly-associated cells [98]. Alternatively, the number of cells can be evaluated using an assay based on conversion of methylthiazole tetrazolium (MTT) to a colored formazan product. Use of this approach suggested that the Aa23 doubling time increases from 2 to 3 days as the cells approach stationary phase, and that addition of tetracycline to suppress *Wolbachia* replication does not improve the doubling time [99].

Difficulties in obtaining accurate cell counts and enumerating *w*AlbB in Aa23 cells led us to transfer *w*AlbB from Aa23 cells into a TK-6 thymidine kinase-deficient derivative of C7-10 cells [55]. Because they are resistant to 5-bromodeoxyuridine (BrdU) [100], we reasoned that TK-6 cells provided a means of selecting against Aa23 cells, if donor cells are present in the infected culture. Although selection was not needed, the *w*AlbB infection was lost after five months in TK-6 cells. Nevertheless, analysis of radiolabeled protein profiles in *w*AlbB-infected TK-6 cells led to the observation that the ubiquitin/proteasome pathway may play an important role in the interaction between *Wolbachia* and its host cell [55], and contributed to later work elucidating the roles of a two-gene operon encoding CidA/CidB proteins in cytoplasmic incompatibility [74,75,76].

In its native host, the planthopper *Laodelphax striatellus*, *w*Stri causes CI, as does *w*Pip in *Cx. pipiens* mosquitoes. Although we have had little success with *w*Pip, transfer of *w*Stri from NIAS-AeAl-2 cells to C7-10 cells was highly successful [53], and established a long-term, stable infection with which we have worked for several years. On two separate occasions, however, C/*w*Stri1 cells lost the infection after approximately 130 subcultures, suggesting that with continued passage, the cells suppress *Wolbachia*. Factors that affect stability, such as changes in the ratio of *Wolbachia* to host cells over time, remain to be systematically examined. These observations support the possibility that there is an unknown fitness cost to maintaining *Wolbachia* in cell lines, consistent with the absence of *Wolbachia* in long-established *Ae. albopictus* lines. As new *Wolbachia*-infected lines are developed, investigators should bear in mind the importance of periodically storing cells in liquid nitrogen to recover early-passage cultures, against which changes over time can be evaluated.

**Figure 1:**
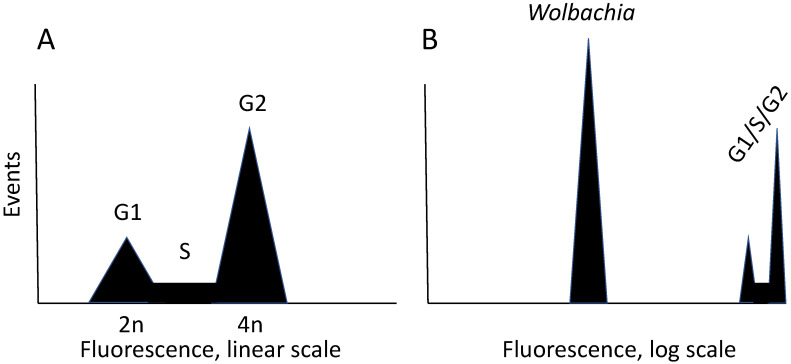
Use of a flow cytometer for evaluating *Wolbachia* abundance. Flow cytometry involves staining a subcellular component, such as DNA, with a fluorescent dye. For DNA, propidium iodide is commonly used. In the instrument, stained cells flow past a detector that records the dye amount, which is displayed on a histogram. Panel (**A**) shows a schematic representation of a typical image obtained with a growing culture of cells. In a mixed population of cells, individual cells independently traverse the cell cycle. Immediately after mitosis, a cell has a diploid content of DNA, which is called G1. As DNA is synthesized, DNA content increases. This increase is represented by the S (synthesis) phase of the cycle. When synthesis is complete, cells have a G2 DNA content. The fluorescence in G2 is double that of G1, as is the total amount of DNA in a cell. In a cell cycle histogram, the *X*-axis is on a linear scale, and areas under the curves are proportional to the percentage of the population in that particular phase of the cycle. (**B**). To detect *Wolbachia* in the same sample, the position of host cell cycle events is electronically shifted to the far right of a histogram with the *X*-axis on a log scale, allowing particles with lower amounts of DNA to be detected. Details of conditions for visualizing *Wolbachia* by flow cytometry are described elsewhere [83].

A major advance in our ability to work with *Wolbachia* in cultured cells is flow cytometry, which allows simultaneous evaluation of cell and bacterial particles (Figure 1).

Using this approach, we have determined that the most infectious *Wolbachia* are those newly-released from fragile host cells, which constitute about 1% of the total *Wolbachia* population in C/*w*Stri1 cells. Establishment of a robust level of *Wolbachia* in a naive cell line requires about 6 days, with an infection ratio of 80 to 100 bacteria per cell. Reminiscent of Aa23 cells, mild aggregation of recipient cells is a typical response of C7-10 cells to infection [56]. Current experiments suggest that *w*Stri replicates better in stationary, as opposed to growing, cells, and we have recently found that high yields of infectious *Wolbachia* can be recovered from mitotically inactivated feeder layers generated by treatment with mitomycin C [101]. In addition to determining conditions that are favorable to establishment of new infections, developing a cell line in which the *Wolbachia* infection itself confers a selective advantage will constitute an important resource for recovery of genetically modified *Wolbachia*.

In addition to changes in the *Wolbachia* phenotype and/or virulence that accompany long-term maintenance outside the wildtype host, much remains to be learned about optimal conditions for maintaining *Wolbachia* in vitro. The relationships between cell cycle parameters, doubling time, and ploidy of cultured cells, relative to conditions that support *Wolbachia* in natural host tissues, have not been systematically explored, nor have effects of nutrient requirements and medium composition. In future studies it will be important to undertake transcriptomic and proteomic analyses to identify genes and gene products that regulate infection and replication of *Wolbachia* in cell lines. Insect cell culture itself remains a developing field, relative to accomplishments that have been achieved with vertebrate cell lines and embryonic stem cells.

## 10. Why Cultured Cells?

If the streamlined *Wolbachia* genome can be genetically engineered in the future, propagation of the altered genome will require efficient reintroduction into a host cell to allow replication and expansion of transformant populations. Use of cell lines offers a practical means of producing the large quantities of *Wolbachia* that will be needed to develop transformation protocols that are sufficiently robust for use in basic research and pest control applications. Although isolated examples of successful transformation of intracellular microorganisms such as *Coxiella burnetti*, the pathogen that causes Q fever, have been achieved, these remain labor intensive and have low frequencies of success [102]. Nevertheless, over the past two decades, remarkable progress towards cell-free culture of *Coxiella* has been achieved, despite its streamlined 2 Mb genome [103,104]. These successes underscore the importance of detailed attention to culture conditions and metabolic activities of obligate intracellular microbes. *Wolbachia* lacks pathogenicity to humans, and its genome is more extensively streamlined, relative to that of *Coxiella*. Nevertheless, the long evolutionary history of *Wolbachia*’s interaction with invertebrate hosts and its adaptations for germline transmission contribute to the value of *Wolbachia* as a model system for understanding the biology of obligate intracellular bacteria in invertebrate cells and manipulating their biology for control of insect pests.

## Figures and Tables

**Table 1:** Cell lines in which *Wolbachia* strains have been propagated.

Cell Line Designation	*Wolbachia* Strain_Supergroup	Source of *Wolbachia*	Reference	Comments
**Dipteran cell lines**				
***Aedes albopictus*** **(mosquito)**				
Aa23	*w*AlbB_B	*Aedes albopictus* embryos	[44]	First infected cell line; established from naturally infected *Ae. albopictus*; one of two *Wolbachia* strains
Aa23(T)	*w*Mel_A	infected RML-12 cells	[45]	12 passages
Aa23(T)	*w*Ri_A *w*Cof_A *w*AlbB_B *w*Pip_B *w*CauA_A *w*CauB_B	*D. simulans* eggs *D. simulans* eggs infected Aa23 cells *Cx. pipiens* eggs *Cadra cautella* eggs *Cadra cautella* eggs	[46]	Demonstration of shell vial technique; details focus on *w*Ri
Aa23(T)	*w*MelPop	*w^1118^* embryos	[43]	Generated *w*MelPop-CLA
NIAS-AeAl-2	*w*Stri_B *w*Kue_A *w*CauA_A	*L. striatellus* ovary *Ephestia kuehniella* eggs *Cadra cautella* eggs	[47]	Infected from small inoculum; one ovary, or 80–100 eggs; Infected AeAl-2 cells form aggregates; occasional addition of uninfected cells to infected cultures
NIAS-AeAl-2	*w*Cau_A *w*CauB_B wKue_A	*Ephestia kuehniella* eggs *Ephestia kuehniella* eggs *Ephestia kuehniella* eggs	[48]	Two stages: infection and maintenance
RML-12	*w*MelPop-CLA_A	infected Aa23 cells	[43]	*w*MelPop transferred to cells; serial passage; reintroduction into original host by microinjection; some loss of virulence; “genetic adaptation” to improve transfer to new hosts
RML-12	*w*Mel_A	O’Neill et al.; cited in [45] personal communication	[45]	Maintained for 3 years
C6/36	*w*Ri_A	*D. simulans* eggs	[46]	
C6/36	*w*Mel_A	infected RML-12 cells	[45]	Stable; higher density than RML-12 cells
C6/36	*w*AlbB_B	infected Aa23 cells	[49]	
C6/36	*w*AlbB_B	infected Aa23 cells	[50]	
C6/36	*w*MelPop-CLA_A	RML-12-CLA	[51]	C6/36.*w*MelPop-CLA
C6/36	*w*AlbB_B	infected Aa23 cells	[52]	Virus screen
C7-10	*w*Stri_B	NIAS-AeAl-2	[53]	Called C/*w*Stri1 line
C7-10	*w*AlbB_B	infected Aa23 cells	[54]	Infected line: C7-10B
C7-10	*w*Ri_A	*D. simulans* eggs	[54]	Infected line: C7-10R C7-10R more stable, uniform than C7-10B
TK-6 (C7-10)	*w*Alb_B	infected Aa23 cells	[55]	Stable 5 months
Mtx-5011-256	*w*Stri_B	C/*w*Stri1 cells	[56]	Lower MOI than C7-10; aneuploidy a factor?
***Aedes aegypti*** **mosquito**				
Aag2	*w*AlbB_B	infected Aa23 cells	[57]	Line called Aag2.*w*AlbB
Aag2	*w*AlbB_B	infected Aa23 cells	[58]	Line called *w*-Aag2
Aag2	*w*Mel_A	*D. melanogaster* embryos	[59,60]	Line called Aag-2*w*Mel
Aag2	*w*Mel_A *w*MelPop-CLA_A	Infected RML-12 cells Infected RML-12 cells	[61]	[43]
Aa-20	*w*MelPop-CLA_A	Not stated	[62]	Mos 20; CVCL_Z353; [63]
***Anopheles gambiae*** **mosquito**				
Mos-55	*w*MelPop-CLA_A	infected Aa23 cells	[43]	
Sua5B	*w*AlbB_B *w*Ri_A	infected Aa23 cells *D. simulans* eggs	[64]	Best was 1/10^3^ cells infected
***Drosophila melanogaster***				
S2	*w*Ri_A	*D. simulans* eggs	[46]	
S2	strain from Dm2008Wb1cells	infected, *D. melanogaster*	[65]	(from abstract; Russian)
Dm2008Wb1	primary cell culture	infected, *D. melanogaster*	[65]	(from abstract; Russian)
JW-18	*w*Mel-Pop_A	infected, *D. melanogaster*	[41]	Albendazole sulfone inhibits
1182-48	*w*MelPop_A	infected JW-18 cells	[66]	Acentriolar haploid line
S2R+	*w*MelPop_A	infected JW-18 cells	[66]	Tetraploid male cells; higher *Wolbachia* titers
***Lutzomyia longipalpis* (sandfly)**				
LL5	*w*MelPop-CLA_A *w*Mel_A	infected RML-12 cells infected RML-12 cells	[67]	Immune activation unstable; no effect on *Leishmania*
Lulo	*w*MelPop-CLA_A *w*Mel_A (unstable)	infected RML-12 cells infected RML-12 cells	[67]	
***Culicoides sonorensis*** **(Biting midge)**				
W3	*w*AlbB_B	infected Aa23 cells	[68]	Line W3
W8	*w*AlbB_B	infected Aa23 cells	[68]	Higher density than W3
***Hematobia irritans*** **(Horn fly)**				
HIE-18	*w*AlbB_B *w*Mel_A *w*MelPop_A	infected Aa23 cells infected Aag2 cells infected Aag2 cells	[69]	50 passages
**Lepidopteran**				
BCIRL-HZ-AM1-G5 *Heliothis zea*	*w*Stri_B	*L. striatellus* ovary	[47]	
Sf9 *Spodoptera frugiperda*	*w*Ri_A	*D. simulans* eggs	[46]	
Sf9 *Spodoptera frugiperda*	*w*CauB_B	*Ephestia kuehniella* eggs	[48]	
**Tick**				
*Ixodes scapularis*	*w*AlbB_B, *w*Stri_B *w*Cfe_F	infected mosquito cells cat fleas	[42]	*w*Stri_B, 29 passages *w*Cfe_F, 2 passages
*Ixodes ricinus*	*w*AlbB_B, *w*Stri_B	infected mosquito cells	[42]	
*Riphicephalus microplus*	*w*AlbB_B, *w*Stri_B	infected mosquito cells	[42]	
**Mammal**				
L929 (mouse)	*w*Stri_B	*L. striatellus* ovary	[47]	Cells maintained at 28 °C
**Filarial screening**				
Aa23	*w*AlbB_B		[39]	Anti-filarial screen
C6/36	*w*AlbB_B	infected Aa23 cells	[40]	Macrofilaricides
JW-18	*w*MelPop_A	*D. melanogaster w^1118^*	[41]	Anti-filarial screen

Insect cell lines in which *Wolbachia* has been maintained. Columns from left to right show: (1) cell lines, arranged in groups according to species from which the cell line was derived; (2) *Wolbachia* strain_supergroup; (3) source of the material introduced into the cell line; (4) reference; (5) brief comments.

## Data Availability

Data described in this review article are available in referenced publications.
